# Hepatic transcript profiling in beef cattle: Effects of feeding endophyte-infected tall fescue seeds

**DOI:** 10.1371/journal.pone.0306431

**Published:** 2024-07-26

**Authors:** Gastón F. Alfaro, Valentino Palombo, MariaSilvia D’Andrea, Wenqi Cao, Yue Zhang, Jonathan E. Beever, Russell B. Muntifering, Wilmer J. Pacheco, Soren P. Rodning, Xu Wang, Sonia J. Moisá

**Affiliations:** 1 Department of Animal Sciences, Auburn University, Auburn, AL, United States of America; 2 Department of Agricultural, Environmental and Food Sciences, Università degli Studi del Molise, Campobasso, Italy; 3 Department of Pathobiology, College of Veterinary Medicine, Auburn University, Auburn, AL, United States of America; 4 Department of Animal Sciences, University of Tennessee, Knoxville, TN, United States of America; 5 Department of Poultry Sciences, Auburn University, Auburn, AL, United States of America; 6 HudsonAlpha Institute for Biotechnology, Huntsville, AL, United States of America; Universitat Jaume 1, SPAIN

## Abstract

The objective of our study was to evaluate the effect of endophyte-infected tall fescue (E^+^) seeds intake on liver tissue transcriptome in growing Angus × Simmental steers and heifers through RNA-seq analysis. Normal weaned calves (~8 months old) received either endophyte-free tall fescue (E^-^; n = 3) or infected tall fescue (E^+^; n = 6) seeds for a 30-d period. The diet offered was *ad libitum* bermudagrass (Cynodon dactylon) hay combined with a nutritional supplement of 1.61 kg (DM basis) of E^+^ or E^-^ tall fescue seeds, and 1.61 kg (DM basis) of energy/protein supplement pellets for a 30-d period. Dietary E^+^ tall fescue seeds were included in a rate of 20 μg of ergovaline/kg BW/day. Liver tissue was individually obtained through biopsy at d 30. After preparation and processing of the liver samples for RNA sequencing, we detected that several metabolic pathways were activated (i.e., upregulated) by the consumption of E^+^ tall fescue. Among them, oxidative phosphorylation, ribosome biogenesis, protein processing in endoplasmic reticulum and apoptosis, suggesting an active mechanism to cope against impairment in normal liver function. Interestingly, hepatic protein synthesis might increase due to E^+^ consumption. In addition, there was upregulation of “thermogenesis” KEGG pathway, showing a possible increase in energy expenditure in liver tissue due to consumption of E^+^ diet. Therefore, results from our study expand the current knowledge related to liver metabolism of growing beef cattle under tall fescue toxicosis.

## Introduction

Tall fescue (*Schedonorus arundinaceus* (Schreb.) Dumort.) is the predominant cool-season forage in the southeastern region of the United States due to its excellent productive characteristics. However, the superlative aptitude of tall fescue is based on the symbiotic relationship with a fungal endophyte called *Epichloé coenophiala* [1]. Ergot alkaloids are produced as secondary metabolites by the fungus. The consumption of ergot alkaloids causes numerous harmful effects on cattle health and performance. Ergot alkaloids, especially ergovaline, bind to monoamine neurotransmitter receptors binding sites (i.e., dopamine, serotonin, etc.), acting as activators and inhibitors in the anterior pituitary. The mimicking effect of ergovaline on monoamine receptors can cause the inhibition of different hormones such as prolactin, adrenocorticotropic hormone (ACTH), and follicle-stimulating hormone (FSH). For example, dopamine is a neurotransmitter that can bind different types of dopamine receptors, which are different depending on the tissue. The inhibition of prolactin occurs by dopamine through binding the dopamine receptors located in the lactotropic cells of the anterior pituitary. Dopamine-receptor 2 (DRD2) is present in the anterior pituitary and is coupled to a Gα protein that inhibits cAMP after dopamine coupling [2]. Ergovaline not only can bind to DRD2 but also is able to inhibit cAMP production in a similar manner compared to dopamine *in vitro* [3, 4].

Animals consuming ergot-contaminated grains or toxic endophyte-diet experience manifest alterations in liver metabolism because of detoxification processes. Notably, beef steers exposed to high E^+^ tall fescue showed that genes related to ATP synthesis, proline and serine, and pyruvate formation were upregulated in steers consuming high-endophyte fescue. These results indicate that the exposure to high-toxic fescue diets upregulates genes involved in energy metabolism [5]. Similarly, mice receiving E^+^ diets had an upregulation of hepatic expression of ATP synthase H^+^ transporting gene (*ATP5b*) which could be related to a feedback mechanism of hepatocytes due to a reduction in cholesterol levels in animals exposed to ergot alkaloids [6]. However, this increase in ATP synthesis capacity by the liver might be a compensatory response to the greater need for meeting energy demands in the condition of a reduction of liver size due to fescue toxicosis occurrence [7]. Similarly, an upregulation of CYP isoforms, a set of genes that codify for proteins involved in the cytochrome P450 system, and a downregulation of genes that codify for antioxidant enzymes in rats [8].

Beef steers grazing E^+^ have greater rectal and skin temperature, lesser average daily gain, and lesser serum prolactin levels compared to animals grazing E^-^ tall fescue [9]. In addition, the cost of E^-^ tall fescue is also greater compared with E^+^, causing a difficulty in the adoption by producers, especially if the infestation rate is not high [10].

Previous reports from microarray data, indicate that consumption of ergot alkaloids by animals grazing E^+^ pastures causes changes in the liver transcriptome [5, 6]. Thus, the main objective of our study was deepening the knowledge of the effects of E^+^ on liver transcriptome metabolism of growing beef cattle consuming E^-^ vs. E^+^ tall fescue seeds with known concentrations of ergovaline, using RNA-seq.

## Materials and methods

### Animals and experimental design

**Fig 1:**
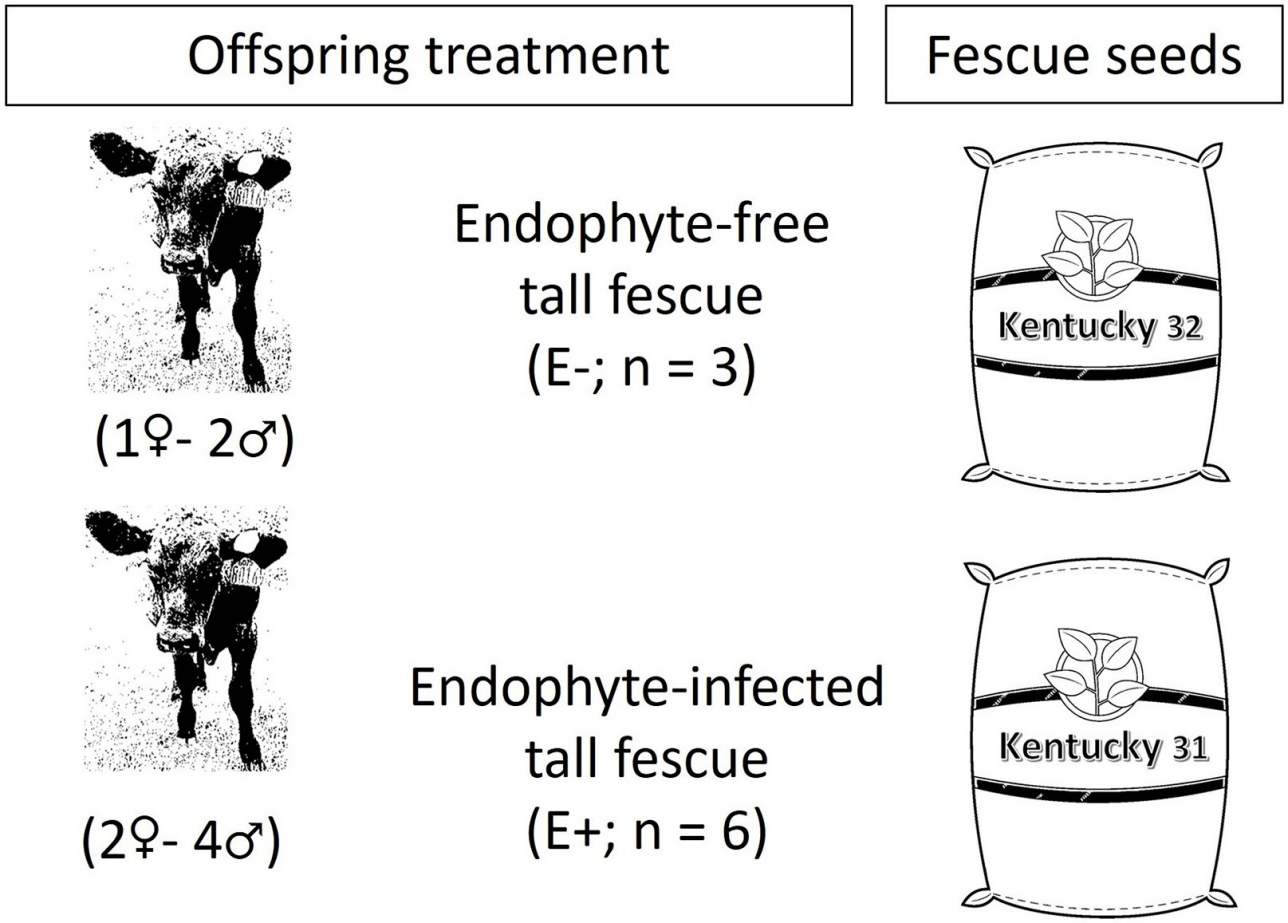
Experimental design.

All the procedures for this study were conducted following a protocol approved by the Institutional Animal Care and Use Committee of Auburn University (IACUC Protocol #2019–3484). Mature Angus × Simmental cows and heifers were the dams of the animals used in this study. These dams were a subset of a group of beef cows located at Black Belt Research Center (32°28’16.32"N 87°13’54.12"W, Marion Junction, Alabama) belonging to Auburn University, Auburn, AL. Detailed description of the experimental design can be found in our previous publication [11]. From these mentioned dams’ offspring, a group of 9 Angus × Simmental weaned steers (n = 6) and heifers (n = 3) with average body weight (BW; 331 ± 36 kg) and age of 7–9 months old were utilized and allocated in two groups based on dietary treatment: 1) Endophyte-infected tall fescue (E^+^; n = 6), and 2) Endophyte-free tall fescue (E^-^; n = 3). There was a steer:heifer ratio of 2:1 in all treatments (e.g., E^+^ = 4 steers and 2 heifers; E^-^ = 2 steers and 1 heifer; Fig 1).

The diet offered was *ad libitum* bermudagrass (Cynodon dactylon) hay combined with a nutritional supplement composed on average of 1.61 kg (on a dry matter (DM) basis) of E^+^ or E^-^ tall fescue seeds, and 1.61 kg (DM basis) of pellets. The pellets were composed of 46.5% ground corn, 46.5% soybean meal, 5% wheat middlings, and 2% soybean oil, and 0.1 kg of molasses per animal per day (S1 Table). The diet was formulated to meet animal nutrient requirements [12] and it was offered twice per day (S2 Table). In the E^+^ group, tall fescue seeds were offered based on their actual ergovaline concentration. Ergot alkaloids concentration of the seeds offered was measured at the Veterinary Medical Diagnostic Laboratory at the University of Missouri (Columbia, MO). There were two lots of tall fescue seeds used in this study, with an ergovaline concentration of 7300 ppb and 2700 ppb, respectively. A total of 20 μg of ergovaline/kg BW/day was the daily dietary individual dose of ergovaline to E^+^ steers and heifers. This pharmacological ergovaline concentration follows the recommendations from previous studies [13, 14] for ensuring the occurrence of fescue toxicosis. Prolactin analysis was performed in offspring serum samples using a prolactin enzyme immunoassay kit (Arbor Assays, Michigan, USA) using a dilution factor 1:100. Offspring performance data is presented in S1–S3 Figs.

### Liver biopsies

After using an ultrasound machine to identify the optimal area to perform the liver biopsy, 5 mL of Lidocaine 2% (VetOne®, Boise, ID) were injected to eliminate any pain during the biopsy procedure. Liver samples (0.5–1 g) were obtained at the end of the treatment period (i.e., 30 days after the beginning of the study), using a sterilized bone marrow aspiration needle (Monoject™, Dublin, Ireland) [15]. For complete information about liver biopsies please see our companion paper [16]. Furthermore, animals were monitored for body weight, rectal temperature, respiration rate (breaths/minute) and hair shedding score. Hair scores were determined by a trained observer on a weekly basis during the 30-day trial. This data is presented in our previous publication [11].

### RNA extraction and library construction

The total RNA of liver samples was extracted using the ZYMO Quick DNA/RNA Miniprep Plus Kit (Zymo Research, CA). RNA integrity of the samples was above 8 (RIN > 8.0) in general. RNA sequencing libraries were constructed using the NEBNext Ultra II Directional RNA Library Prep Kit for Illumina (New England Biolabs, MA) with a 1500 ng total RNA input. The libraries were sequenced on an Illumina NovaSeq 6000 instrument to generate 150-nucleotide paired-end reads. For a complete description see companion paper [16].

### RNA-seq and differential gene expression analysis

A detailed description of the RNAseq analysis and the bioinformatics analysis could be found in our companion paper [16]. Briefly, a total number of 794,813,112 read pairs were generated for the nine transcriptomes, with sequencing yields ranging from 75,864,914 to 108,428,902 reads per sample. For RNAseq yield see S3 Table. The read quality was checked by FastQC v11.5 [17]. Sequencing adapter sequences and low-quality bases were trimmed using Trimmomatic v0.36 [18]. On average, 98.47% of reads survived quality filtering, and these high-quality reads were mapped to the cattle reference genome (GenBank: GCA_002263795.2) by Tophat-2.1.1 [19, 20]. The average mapping percentage is 86.52% (S2 Table). RNA concentration was 887 ± 157.45 ng/uL. Our objective was to detect DEGs exclusively expressed in animal consuming E^+^ or E^-^ tall fescue seeds. Therefore, E^+^ vs. E^-^ groups were compared to characterize the liver transcriptomic profile under E^-^ or E^+^ seeds supplementation. Additionally, 14 DEGs (S4 Table) were selected for qRT-PCR validation (S4 Fig) based on the DEGs with greater expression values. For extended information about qRT-PCR validation, please see companion paper [16].

### Functional annotation of genes

Database for Annotation, Visualization, and Integrated Discovery (DAVID, version 6.8) [21] was used for functional annotation. DAVID assigned genes to pathways as per the Kyoto Encyclopedia of Genes and Genomes (KEGG), and determined enrichment of pathways using Fisher’s exact test [22]. In order to account for multiple testing, a Benjamini-Hochberg correction was applied [23]. A list of DEG was generated using FDR < 0.05 as a cutoff value (S9 Table). Pathways were deemed significant if they obtained a corrected *p*-value of < 0.05. Pathways specifically addressing human diseases and disorders were not included in further analysis of DAVID identified pathways, as these were not relevant to this study.

### Dynamic impact approach

**Fig 2:**
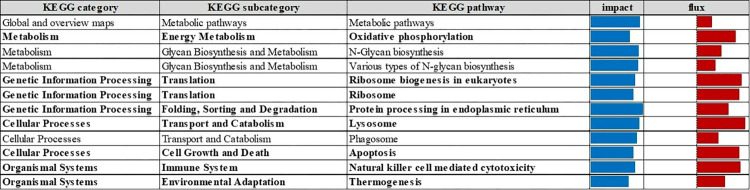
Summary of flux and impact results identified by the Dynamic Impact Approach (DIA) based on Kyoto Encyclopedia of Genes and Genomes (KEGG) pathways databases analysis of the bovine liver transcriptome of growing beef cattle supplemented with E^+^ and E^-^ fescue seeds. Footnotes: flux represents the direction of each category and the corresponding subcategory: red color shows activation. Blue lines show the impact of each category and the corresponding subcategory (P value < 0.05; FDR < 0.05). Subcategories and pathways highlighted in bold met the defined cutoff criteria for discussion.

We utilized the Dynamic Impact Approach (DIA) analysis for estimating the impact and flux of all the manually curated pathways associated with the KEGG database [24]. We defined the term ‘impact’ as the change in the expression of the genes belonging to a specific pathway due to the supplementation of E^+^; and ‘flux’ as the report of the average direction in the expression as downregulation, upregulation, or neutral or no change. The entire dataset, including Entrez gene IDs, FDR, Fold Change (FC), and *p*-values of each treatment group (E^+^ and E^-^) were uploaded into DIA, and the overall cutoff was applied on FDR and *p*-value < 0.05 as the threshold (Fig 2).

### PANEV visualization analyses

**Fig 3:**
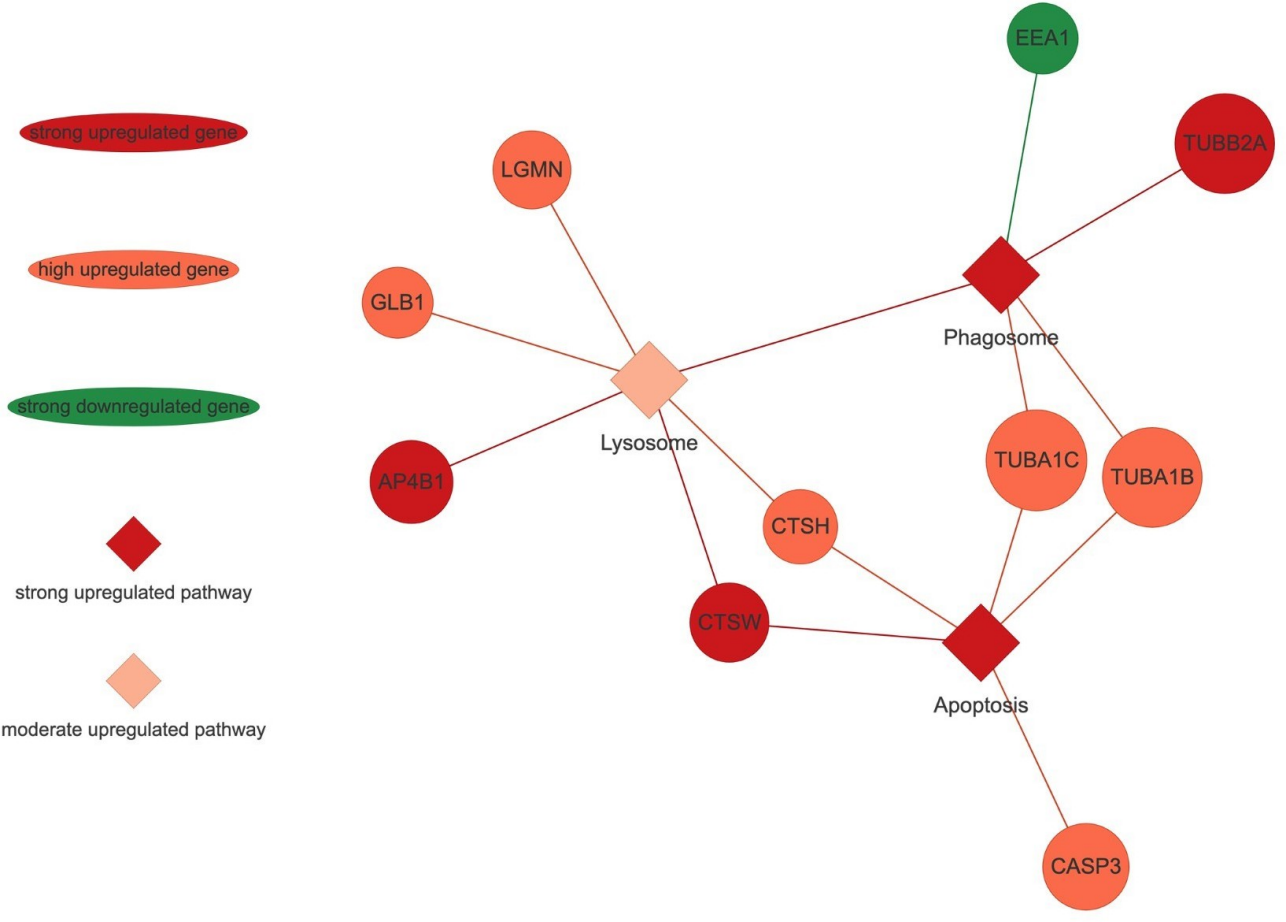
PANEV visualization of ‘Cellular processes’ KEGG category. Footnotes: circles, rhombuses, and lines in dark green color represent strong downregulation of the specific pathway or gene. Pink color represents a low upregulated gene.

**Fig 4:**
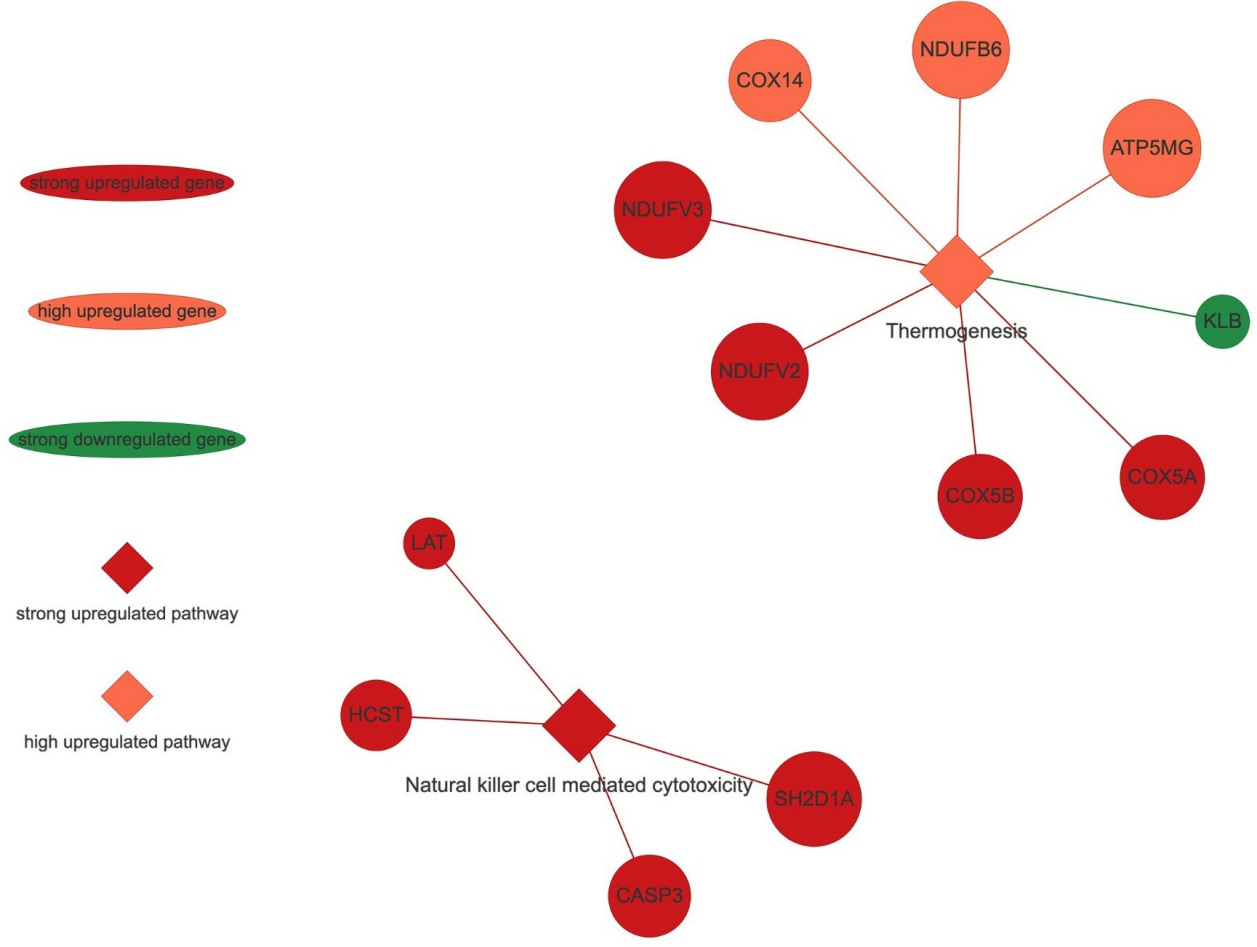
PANEV visualization of ‘Organismal systems’ KEGG category. Footnotes: circles, rhombuses, and lines in green color represent downregulation of the specific pathway or gene, whereas those in red color represent upregulation.

The PANEV (Pathway Network Visualizer) tool [25] has been used to visualize the results in a context of gene/pathway networks, and pinpoint candidate genes associated with a subset of pathways of interest. PANEV v.1.0 is an R package which utilizes KEGG database to retrieve information about each KEGG pathway. This method helped us to visualize the interconnection among key genes and KEGG pathways that were significantly impacted by the treatment applied (Figs 3 and 4, S4 and S5 Figs).

## Results

This publication focuses on the effect of feeding endophyte and endophyte-free fescue seed. Our companion paper focuses on the effect of feeding rumen-protected niacin [16]. The results presented in these publications were generated from the same research project. During the analysis of our RNAseq data, we detected 1131 DEG due to rumen-protected niacin supplementation and 758 DEG due to fescue seed supplementation (FDR < 0.05). When assessing Kegg pathways between both comparisons, we detected several identical results. Therefore, we decided to discuss the 203 DEG exclusively expressed in the E^+^ vs. E^-^ tall fescue comparison (S7 Fig), considering DAVID and DIA functional analysis [24]. Overall, among the 203 DEGs a total of 153 genes resulted upregulated with a range of log2 fold change from 1.836 to 5.055, and 50 genes were downregulated with a range of log2 fold change from -2.058 to -9.389. The top 10 up- and down-regulated DEGs (FDR ≤ 0.05) are listed in S5 Table. Regarding the functional analysis, an upregulated expression pattern was detected for several KEGG pathways using DIA functional analysis (Fig 2). It is important to highlight the prevalence of upregulated genes compared to downregulated ones. This determines the flux of each KEGG pathway. Therefore, no pathways with fluxes that tend to downregulation are present in Fig 2. In contrast, no Gene Ontology terms resulted significantly enriched (FDR ≤ 0.05; S6–S8 Tables). The cutoff criteria for selecting relevant KEGG results for discussion was to consider those KEGG subcategories and KEGG pathways that met two cutoffs: a) having a value higher than 0.6 of the difference between the absolute value of flux and the impact value and, b) having an impact value greater than 50% of the maximum total impact. Almost all representative KEGG categories (i.e., ‘Metabolism’, ‘Genetic Information Processing’, ‘Cellular processes’, and ‘Organismal system’) were impacted by fescue seed supplementation showing, in general, an activation (or up-regulation) (3 and 4, S5 and S6 Figs respectively). The KEGG categories ‘Global and overview maps’ and ‘Environmental information processing’ did not have any significantly impacted KEGG subcategory according to our established cutoff criteria; therefore, they were not considered in the discussion. KEGG pathways that met our cutoff criteria appear bolded in Fig 2.

### Metabolism

The KEGG category “Metabolism” had significant activation of the KEGG subcategory ‘Energy Metabolism’ (Fig 2). Within the ‘Energy Metabolism’ KEGG subcategory, the ‘Oxidative phosphorylation’ KEGG pathway had a significant activation due to the upregulation of the genes NADH:Ubiquinone Oxidoreductase Core Subunit V2 (*NDUFV2*, logFC = 1.14; *p* = 0.002), NADH:Ubiquinone Oxidoreductase Core Subunit V3 (*NDUFV3*, logFC = 1.41; *p* = 0.0002), NADH:Ubiquinone Oxidoreductase Subunit B6 (*NDUFB6*, logFC = 1; *p* = 0.003), Cytochrome C Oxidase Subunit 5A (*COX5A*, logFC = 1.12; p = 0.0003), Cytochrome C Oxidase Subunit 5B (*COX5B*, logFC = 1.13; *p* = 0.0006), and ATP Synthase Membrane Subunit G (*ATP5MG*, logFC = 1; *p* = 0.0007) (S5 Fig).

### Genetic information processing

Within the ‘Genetic Information Processing’ KEGG category, the KEGG subcategory ‘Translation’ has a significant activation of the KEGG pathways ‘Ribosome biogenesis in eukaryotes’ and ‘Ribosomes’ (Fig 2). The ‘Ribosome biogenesis in eukaryotes’ KEGG pathway was significantly activated due to the upregulation of the genes NIN1 (RPN12) Binding Protein 1 Homolog (*NOB1*, logFC = 1.75; *p* = 7.19 x 10^−6^), Casein Kinase 2 Alpha 1 (*CSNK2A1*, logFC = 1.07; *p* = 0.0008), G Protein Nucleolar 2 (*GNL2/NUG2*, logFC = 0.98; *p* = 0.002), and N-Acetyltransferase 10 (*NAT10/KRE33*, logFC = 0.96; *p* = 0.002). The ‘Ribosome’ KEGG pathway was significantly activated due to the upregulation of the genes Ribosomal Protein S3 (*RPS3*, logFC = 1.40; *p* = 5.14 x 10^−5^), Mitochondrial Ribosomal Protein L27 (*MRPL27*, logFC = 1.36; *p* = 9.9 x 10^−5^), Ribosomal Protein S16 (*RPS16*, logFC = 1.26; *p* = 0.0005), Ribosomal Protein S5 (*RPS5*, logFC = 1.20; *p* = 0.0004), Ribosomal Protein L23a (*RPL23A*, logFC = 0.97; *p* = 0.001), Mitochondrial Ribosomal Protein L11 (*MRPL11*, logFC = 0.96; *p* = 0.002) and, Mitochondrial Ribosomal Protein L24 (*MRPL24*, logFC = 0.95; *p* = 0.002). Furthermore, also within the ‘Genetic Information Processing’ KEGG category, the KEGG subcategory ‘Folding, Sorting and Degradation’ has a significant activation of the KEGG pathway ‘Protein processing in endoplasmic reticulum’ (Fig 1). This KEGG pathway had a significant activation due to the upregulation of the genes Ring-Box 1 (*RBX1*, logFC = 1.63; *p* = 5.77 x 10^−6^), E3 ubiquitin-protein ligase RBX1 (*LOC780968*, logFC = 1.45; *p* = 1.04 x 10^−5^), Protein Disulfide Isomerase Family A Member 6 (*PDIA6*, logFC = 1.24; *p* = 0.0003), Membrane Associated Ring-CH-Type Finger 6 (*MARCHF6/DOA10*, logFC = 1.18; *p* = 0.003), Ribophorin II (*RPN2*, logFC = 0.97; *p* = 0.002) and, Ribophorin I (*RPN1/OST1*, logFC = 0.94; *p* = 0.003). In contrast, Mannosidase Alpha Class 1A Member 2 (*MAN1A2*, logFC = -1.94; *p* = 0.0004) was downregulated (S6 Fig).

### Cellular processes

Within the ‘Cellular Processes’ KEGG category, the KEGG subcategory ‘Transport and Catabolism’ has a significant activation of the ‘Lysosome’ KEGG pathway and, the KEGG subcategory ‘Cell Growth and Death’ has a significant activation of the ‘Apoptosis’ KEGG pathway (Fig 2). The activation of the ‘Lysosome’ KEGG pathway was due to the upregulation of the genes Cathepsin W (*CTSW*, logFC = 1.76; *p* = 9.83 x 10^−5^), Adaptor Related Protein Complex 4 Subunit Beta 1 (*AP4B1*, logFC = 1.52; *p* = 0.0002), Cathepsin H (*CTSH*, logFC = 1.24; *p* = 0.001), Legumain (*LGMN*, logFC = 1.21; *p* = 0.001), and Galactosidase Beta 1 (*GLB1*, logFC = 1.04; *p* = 0.002). Furthermore, the activation of the ‘Apoptosis’ KEGG pathway was due to the upregulation of the genes *CTSW*, *CTSH*, Caspase 3 (*CASP3*, logFC = 1.21; *p* = 0.003), Tubulin Alpha 1c (*TUBA1C*, logFC = 1.14; *p* = 0.001) and Tubulin Alpha 1b (*TUBA1B*, logFC = 1.02; *p* = 0.002) (Fig 3).

### Organismal systems

Finally, within the ‘Organismal Systems’ KEGG category, the KEGG subcategory ‘Immune System’ has a significant activation of the ‘Natural killer cell mediated cytotoxicity’ KEGG pathway, whereas the KEGG subcategory ‘Environmental Adaptation’ has a significant activation of the ‘Thermogenesis’ KEGG pathway (Fig 2). The ‘Natural killer cell mediated cytotoxicity’ KEGG pathway presented the activation of the genes SH2 Domain Containing 1A (*SH2D1A*, logFC = 1.52; *p* = 0.001), Hematopoietic Cell Signal Transducer (*HCST/DAP10*, logFC = 1.51; *p* = 0.002), Linker for Activation of T cells (*LAT*, logFC = 1.38; *p* = 0.001) and *CASP3*. The ‘Thermogenesis’ KEGG pathway had activation of the genes *NDUFV3*, *NDUFV2*, *COX5B*, *COX5A*, *ATP5MG*, *NDUFB6*, Cytochrome C Oxidase Assembly Factor COX14 (*COX14*, logFC = 0.95; *p* = 0.003) and down-regulation of the gene Klotho Beta (*KLB*, logFC = -1.41; *p* = 0.003) (Fig 4).

## Discussion

### Metabolism

Oxidative phosphorylation is a metabolic pathway occurring in the inner membrane of the mitochondria of eukaryote organisms. As a result of this process, chemical energy in the form of ATP is released due to the exchange of electrons from different molecules [26]. In our study, we observed an upregulation in three genes that codify for subunits belonging to Complex I or NADH:Ubiquinone Oxidoreductase, such as *NDUFV2*, *NDUFV3*, and *NDUFB6*. In addition, genes involved in Complex IV or Cytochrome c oxidase, such as Cytochrome C Oxidase Subunits 5 A and B (*COX5A* and *COX5B*, respectively), and ATP Synthase Membrane Subunit G (*ATP5MG*) showed an upregulation in cattle consuming E^+^. Coincidentally, a previous study also detected an upregulation of genes involved in the oxidative phosphorylation pathways in liver tissue on beef steers grazing high endophyte-infected tall fescue compared with those exposed to low endophyte-infected tall fescue [5]. These results are congruent with ours, suggesting a possible collective activation of energy-related genes by ergot alkaloids. One plausible mechanism of action could be associated with a greater demand for ATP production by hepatic cells to sustain their normal metabolism. Our results suggest a possible activation of mitochondrial potential for ATP generation by ergot alkaloids due to an increased physiological requirement for ATP in the liver of growing beef cattle consuming E^+^ fescue seeds. This response was also noticed in a previous study [5]. However, the mitochondrial ATP synthase could also work in the direction of ATP hydrolysis when mitochondria are deprived of oxygen and the membrane potential decreases [27]. Remarkably, ATP synthase has an important role in the permeabilization of the inner mitochondrial membrane to low molecular weight solutes [28] and in the formation of mitochondrial mega-channels (i.e., permeability transition pore) [29]; which leads to apoptosis [30]. Our results showed an apoptosis activation by E^+^ fescue seeds consumption. In other words, our data present signs of potential instability of hepatocytes mitochondrial membrane by E^+^ seeds supplementation that could lead to apoptosis.

### Genetic information processing

Genes differentially expressed present in the ‘Folding, Sorting and Degradation’ KEGG subcategory give signs of activation of RING finger domain of proteins present in the ubiquitin ligase complex at the cytoplasm level (*RBX1*) and, at the endoplasmic reticulum membrane level (*MARCHF6*). These genes are suspected of presenting a scaffolding function in engaging and positioning E2 and substrate for Ub transfer during the ubiquitination process that leads to proteasome degradation [31].

Sequestration of critical cellular chaperones and vital transcription factors by misfolded proteins is one of the typical effects of a toxicity response [32]. In our study, this response was characterized by the activation of *RPN1* and *RPN2*, which are components of the largest subunit of 26S proteasome. Thus, *RPN1* acts as a chaperone that recognizes misfolded proteins and *RPN2* has a role in translocation and the maintenance of the structure of the rough endoplasmic reticulum [33]. Furthermore, E^+^ fescue seeds also upregulate *PDIA6*, which acts as a chaperone that inhibits the aggregation of misfolded proteins [34]. Therefore, the activation of the ‘Protein processing in the endoplasmic reticulum’ KEGG pathway lead us to suggest that E^+^ fescue seeds might produce an accumulation of unfolded proteins in the endoplasmic reticulum lumen due to a disturbance in the endoplasmic reticulum’s redox state [35]. The activation of unfolded protein response sensors is required to alleviate the effects of endoplasmic reticulum stress. Although, more investigation into this statement should be addressed.

Hepatocytes could potentially need to rebuild the degraded proteins caused by the exposure to elevated ergot alkaloids [36]. Consequently, our study showed an upregulation of the protein synthesis machinery: the ribosome. The ‘Ribosome biogenesis in eukaryotes’ KEGG pathway is strongly related to cell growth, cell division, and cell regeneration [37]. Interestingly, our study showed an upregulation in *NOB1*, an anti-apoptotic gene in eukaryotes, involved in synthesis and degradation of proteins, and *RPN12*, a key regulator of proteasome integrity [38]. However, since there was an activation on ‘Apoptosis’ KEGG pathway, *NOB1* activation may be related to the protein synthesis necessary for ribosome biogenesis more than the cellular anti-apoptosis function. Similarly, *NAT10* gene codifies for a protein involved into RNA acetyltransferase, which promotes mRNA translation in eukaryotic organisms [39]. Collectively, the upregulation of genes related to protein synthesis and ribosome biogenesis suggests that consumption of E^+^ diet could have a stimulatory effect of protein synthesis in hepatic cells. Although, careful should be exercised with this statement because it could suggest that we are facing accumulation of aggregation-prone proteins that could be harmful to cells.

Furthermore, another sign for the increase in protein synthesis by the consumption of an E^+^ diet was the upregulation of genes related to the “Ribosome” pathway, such as *RPS3* and *MRLP27*. The 40S Ribosomal Protein family is a small subunit of the ribosome and plays a key role in the eukaryotic ribosomal machinery during translation. For example, *RPS3* is involved in the translation initiation by binding the 40S subunit to eIF1 and eIF1A, enhancing the recognition of the start codon [40]. Similarly, *MRLP27* constitutes one of the several mitochondrial ribosomal proteins involved in phosphorylation activity, and it is located near peptidyl transferase, enzyme responsible for the addition of amino acids in the growing polypeptide chain [41]. Previously, an upregulation in *RPS16* and Ribosomal protein L13A (*RPL13A*) gene expression in liver of male rats exposed to E^+^ diets compared with those receiving E^-^ diets was reported [42]. Overall, we found a pattern of a potential increase in protein synthesis by the upregulation in ribosome-related pathways due to the apoptotic occurrence of ergot alkaloids toxicosis in liver.

Finally, we observed the downregulation of *MAN1A2* gene which is known to be a target of decreased expression by TNFα [43], a pro-inflammatory cytokine with an active role during E^+^ tall fescue exposure caused by endophyte-infected tall fescue [44]. The upregulation of interferon gamma inducible protein 47 (*IFI47*) gene, which was among the top-ten upregulated genes among our DEG list (S5 Table), partially supported this scenario, considering its role in hepatic injury synergizing with TNFα [45].

### Cellular processes

The liver is a unique organ responsible for numerous metabolic, vascular, detoxifying, secretory, and excretory functions. Its uniqueness relies on the capability of being regenerated through hepatocyte proliferation after exposure to injury related to a toxin [46]. During the exposure to E^+^ tall fescue, the liver of different mammalian species experiences a reduction in weight per unit of kg as a result of the numerous detoxification processes, as shown in rats [42, 47] or beef cattle [7]. More specifically, the underlying mechanisms causing liver size reduction could be linked to numerous physiological processes, such as cell death and apoptosis.

Remarkably, our study showed an upregulation in Caspase-3 (*CASP3*). The involvement of *CASP3* in proteolysis and cellular apoptosis in cattle was previously reported [48, 49]. Hepatocytes apoptosis in cattle takes place during stress conditions; for example, a greater *CASP3* activity, three weeks after parturition in dairy cattle, indicates its greater activity during apoptosis [50]. Since our study also showed an upregulation in the Apoptosis KEGG pathway, it is possible to link the greater expression of *CASP3* with a greater protein processing activity in the endoplasmic reticulum that enhances cell degradation. Logically, to maintain overall liver health, homeostasis is a survival strategy.

Lysosomes are membrane-enclosed compartments that contain acid hydrolases used during intracellular digestion of macromolecules [51]. The glycosidase Galactosidase beta 1 (GLB1) was activated in the liver due to E^+^ fescue seeds consumption. GLB1 is proteolytically processed to generate mature lysosomal enzymes [52]. In mature lysosomes, cysteine proteases have a role in the degradation of lysosomal proteins. Our results showed that the presence of ergovaline from the consumption of endophyte-infected tall fescue seeds activates some of these cysteine proteases like Cathepsin W and Cathepsin H (S8 Table). Furthermore, in our study, the up regulation of Legumain (*LGMN*) could potentially lead to the activation of the mentioned cysteine proteases in the mature active lysosome. Therefore, *LGMN* could have a role in the degradation of compounds produced during ergot alkaloids degradation, like ergopeptines; which undergo hepatic degradation or excretion into the intestines as bile [53]. We also detected signs of translocation of targeting proteins from the trans-Golgi network to the endosomal-lysosomal system due to the activation of adaptor related protein complex 4 subunit beta 1 (*AP4B1*) in the liver [51]. We believe that this targeting proteins could be related to the products of degradation of ergovaline.

### Organismal systems

The energy requirements for maintenance increase due to the greater thermoregulatory mechanisms such as accelerated respiration. For example, our results indicate that E^+^ animals experienced an upregulation in ‘Thermogenesis’ pathways, which requires energy expenditure [54]. However, thermogenesis could also be enhanced by the phosphorylation of ADP during the oxidative phosphorylation process [55].

The upregulation of ‘Immune system’ relied on the upregulation of a cluster of genes in ‘Natural killer cell mediated cytotoxicity’ pathway (Figs 2 and 4), such as *CASP3*, *LAT*, *HCST* and *SH2D1A* genes. Natural killer (NK) cells are involved in innate immunity response [56], in addition to lymphocytes, they aid in the development of immunological memory for enhanced responses to subsequent pathogen exposure [57]. The immunomodulatory effect of ergot alkaloids on the activation of NK cells has been long recognized [58, 59], and our results seemed particularly in line with this. Indeed, it has been observed that the engagement of appropriate NK cell membrane receptors by ergot alkaloids causes an enhancement of NK cell-mediated cytotoxic activity *in vitro* [59]. In particular, the ability of alkaloids to activate human *CASP3*, a key effector of apoptosis [60], has been well-described [61]. In this regard, it is interesting to note that it is well documented in literature the ability of prolactin to modulate the cytotoxic activity of NK cells [62–64] and at the same time, it is remarkable to highlight that alkaloids act at dopamine receptors to inhibit prolactin release [65, 66].

The marked upregulation of ‘Environmental Adaptation’ subcategory, relying on the upregulation of ‘Thermoregulation’ pathway (Figs 2 and 4). Overall, this may be compatible with the fact that consumption of E^+^ tall fescue is known to alter thermoregulatory ability in cattle [67, 68] and that an increase in body temperature was observed in steers fed E^+^ fescue hay [69]. The upregulation of this pathway was due to a cluster of genes from the NADH:ubiquinone oxidoreductase subunit (NDUF) family group and from cytochrome c oxidase (COX) family, notably the *COX5A* gene. NDUF genes are known to be crucial for respiration in many aerobic organism [70], whereas *COX5A* protein has been recently described as differentially expressed in poultry liver under heat stress conditions [71]. More in general, these results appeared consistent with the upregulation of ‘Oxidative phosphorylation’ pathway detected in our experiment (Fig 2) and overall may be compatible with the fact that mitochondria are involved in thermogenesis [72, 73]. Furthermore, the downregulation of *KLB* was intriguing considering its involvement with Fibroblast Growth Factor 21 (*FGF21*) to maintain thermoregulation in response to cold [74].

## Conclusion

The negative effects of tall fescue toxicosis in beef cattle performance is widely known; however, our study elucidated specific impact on hepatic tissue transcriptome on growing Angus × Simmental steers and heifers after 30 d of E^+^ intake at a rate of 20 μg of ergovaline/kg BW/day. The consumption of ergot alkaloids usually decreases feed intake and liver mass and metabolism. This last one demonstrated in our results by the upregulation of KEGG pathways related to oxidative phosphorylation, ribosome biogenesis, lysosome, apoptosis, and protein processing in endoplasmic reticulum. Moreover, another critical effect of E^+^ intake is the thermoregulation misbalance, as shown by the activation of ‘Thermogenesis’ KEGG pathway in our study. Nevertheless, caution must be exercised when interpreting the results, since changes in gene expression might not translate to changes in protein expression or activity and thus, may not directly explain phenotypic responses observed with E^+^ seed consumption.

## Supporting information

S1 Table Chemical composition of diet fed to steers and heifers. (DOCX)

S2 Table Hay and supplement offered. (XLSX)

S3 Table Summary of RNA-seq yield, quality control, and alignment percentages. (DOCX)

S4 Table List of primers used for qRT-PCR validation assays. (DOCX)

S5 Table Top 10 up- and down-regulated differentially expressed genes. (DOCX)

S6 Table Biological processes. (XLSX)

S7 Table Cellular components. (XLSX)

S8 Table Molecular functions. (XLSX)

S9 Table DEGs in each KEGG pathway. (XLSX)

S1 Fig Offspring body weight and average daily gain. (TIF)

S2 Fig Offspring rectal temperature, respiration rate and hair score. (TIF)

S3 Fig Offspring serum prolactin. (TIF)

S4 Fig qPCR validation results. (TIFF)

S5 Fig PANEV visualization of ‘Metabolism’ KEGG category. (TIFF)

S6 Fig PANEV visualization of ‘Genetic Information Processing’ KEGG category. (TIFF)

S7 Fig Venn diagram comparing niacin effect vs fescue seed effect DEGs. (TIFF)
