# Supplementary material for: Hepatic transcript profiling in beef cattle: Effects of feeding endophyte-infected tall fescue seeds
Source: PLoS One. 2024 Jul 26;19(7):e0306431. doi: 10.1371/journal.pone.0306431 (PMC11280227; doi:10.1371/journal.pone.0306431)
Supplement: S1 Table — (DOCX) [file pone.0306431.s001.docx]

**S4 Table.** List of primers used for qRT-PCR validation assays.

| Gene | Primer Sequences (5’→3’) |
| --- | --- |
| *APOE* | Forward: GCTGCTCAACACCCAGGTCA |
|  | Reverse: CATGGGGCCTAGCTGTCCCT |
| *AKT2* | Forward: CTGGCGATACTGTCGGACCC |
|  | Reverse: AGTCGGGAGACGGTTTGAGT |
| *ARHGDIA* | Forward: ACATCCAGCACACGTACAGG |
|  | Reverse: GTCGTCTGTGAAGCGGGACT |
| *LRPAP1* | Forward: AGTCAGCCAACTTCACCGAGA |
|  | Reverse: GTGTGGCCTCAGAGCTCGTT |
| *GABARAPL2* | Forward: TTGGTGCGCTGAGCTCCG |
|  | Reverse: TCCACAATCACCCAGCGAGT |
| *HSPB1* | Forward: ACTCGCAAATACACGCTGCC |
|  | Reverse: ACGCGCCTGGAAGGTGAC |
| *NFKBIB* | Forward: AACAAACCCGAGCCCACCTG |
|  | Reverse: GCCACCATACATGCGGACGG |
| *RPS6KB2* | Forward: CCCTCCGTCCTGGATAGCAT |
|  | Reverse: CTGGGCCGGAACCCCTC |
| *PLCB1* | Forward: TCGGACGGACCTTGTCT |
|  | Reverse: CCGTCCAGGAGTGACTGA |
| *TGFB1* | Forward: GGGTCTCCCGAGGAAAAGGT |
|  | Reverse: AGCTCACCCATCTCGGTT |
| *GADD45B* | Forward: ACGTGACAGAGGACCAGT |
|  | Reverse: GGACCTCCCTCTACATCCCT |
| *FASN* | Forward: AGAACGCTGGCCCCACGAA |
|  | Reverse: GGTCGCCCTCGATGATGTGT |
| *HRAS* | Forward: TCCAGCTCCGGGACCCTCT |
|  | Reverse: GGCTCAGCTTGCGCACCT |
| *JUN* | Forward: CAGCCTGAAGCCGCACCTCC |
|  | Reverse: CAGGCCAGCCGCGCCGTA |
