# Supplementary material for: Hepatic transcript profiling in beef cattle: Effects of feeding endophyte-infected tall fescue seeds
Source: PLoS One. 2024 Jul 26;19(7):e0306431. doi: 10.1371/journal.pone.0306431 (PMC11280227; doi:10.1371/journal.pone.0306431)
Supplement: S3 Table — (DOCX) [file pone.0306431.s003.docx]

**S3 Table.** Summary of RNA-seq yield, quality control, and alignment percentages

| Sample_name | Number of reads | Reads after trimming | Trim surviving % | Mapped reads | Mapping % |
| --- | --- | --- | --- | --- | --- |
| 9009G_E+F_0716 | 108428902 | 106895644 | 98.59% | 90941355 | 85.10% |
| 9050G_E+M_0716 | 92735514 | 90642050 | 97.74% | 80530569 | 88.80% |
| 9085G_E+M_0716 | 90696096 | 89180578 | 98.33% | 76712885 | 86.00% |
| 9001G_E+M_0716 | 89668978 | 88728726 | 98.95% | 82738585 | 93.20% |
| 9008G_E+M_0716 | 75864914 | 74663634 | 98.42% | 63083703 | 84.50% |
| 9093G_E+F_0716 | 85047190 | 84004512 | 98.77% | 75555190 | 89.90% |
| 9036G_E-M_0716 | 82169668 | 80461072 | 97.92% | 72578322 | 90.20% |
| 9056G_E-F_0716 | 89534924 | 86789500 | 96.93% | 60555620 | 74.60% |
| 9120G_E-M_0716 | 80666926 | 78998438 | 97.93% | 68225659 | 86.40% |
