# Supplementary material for: Hepatic transcript profiling in beef cattle: Effects of feeding endophyte-infected tall fescue seeds
Source: PLoS One. 2024 Jul 26;19(7):e0306431. doi: 10.1371/journal.pone.0306431 (PMC11280227; doi:10.1371/journal.pone.0306431)
Supplement: S4 Table — (DOCX) [file pone.0306431.s004.docx]

**S5 Table.** Top 10 up- and down-regulated differentially expressed genes.

| **Rank** | **Up-/down-regulation** | **Entrez ID** | **Gene symbol** | **FC** | **P-value** |
| --- | --- | --- | --- | --- | --- |
| 1 | Upregulated | 513767 | *LOC513767* | 5.05 | 1.47E-06 |
| 2 |  | 617313 | *LOC617313* | 5.03 | 2.00E-04 |
| 3 |  | 521987 | *IFI47* | 4.58 | 7.94E-04 |
| 4 |  | 509860 | *S100A2* | 4.25 | 6.88E-04 |
| 5 |  | 281210 | *GPX3* | 4.19 | 1.12E-03 |
| 6 |  | 616423 | *C29H11orf98* | 3.93 | 1.76E-08 |
| 7 |  | 100139898 | *BRICD5* | 3.90 | 3.98E-04 |
| 8 |  | 508633 | *CD8B* | 3.67 | 2.48E-03 |
| 9 |  | 616317 | *STMN1* | 3.61 | 1.35E-05 |
| 10 |  | 510967 | *CTSW* | 3.39 | 9.83E-05 |
| 1 | Downregulated | 281152 | *FASN* | -9.39 | 1.39E-03 |
| 2 |  | 526028 | *SEMA4C* | -7.83 | 3.33E-03 |
| 3 |  | 107131164 | *ADAMTSL5* | -6.88 | 2.13E-03 |
| 4 |  | 540473 | *ASCL1* | -6.86 | 2.60E-03 |
| 5 |  | 539556 | *PDE4D* | -6.67 | 9.13E-04 |
| 6 |  | 533803 | *TCEA3* | -5.80 | 2.27E-03 |
| 7 |  | 505059 | *WTIP* | -5.48 | 2.60E-03 |
| 8 |  | 538535 | *C7H5orf24* | -5.02 | 1.63E-03 |
| 9 |  | 514745 | *DHCR7* | -4.65 | 6.89E-04 |
| 10 |  | 615408 | *SEPTIN5* | -4.42 | 7.37E-05 |
