# Supplementary figures and images for: Hepatic transcript profiling in beef cattle: Effects of feeding endophyte-infected tall fescue seeds
Source: PLoS One. 2024 Jul 26;19(7):e0306431. doi: 10.1371/journal.pone.0306431 (PMC11280227; doi:10.1371/journal.pone.0306431)

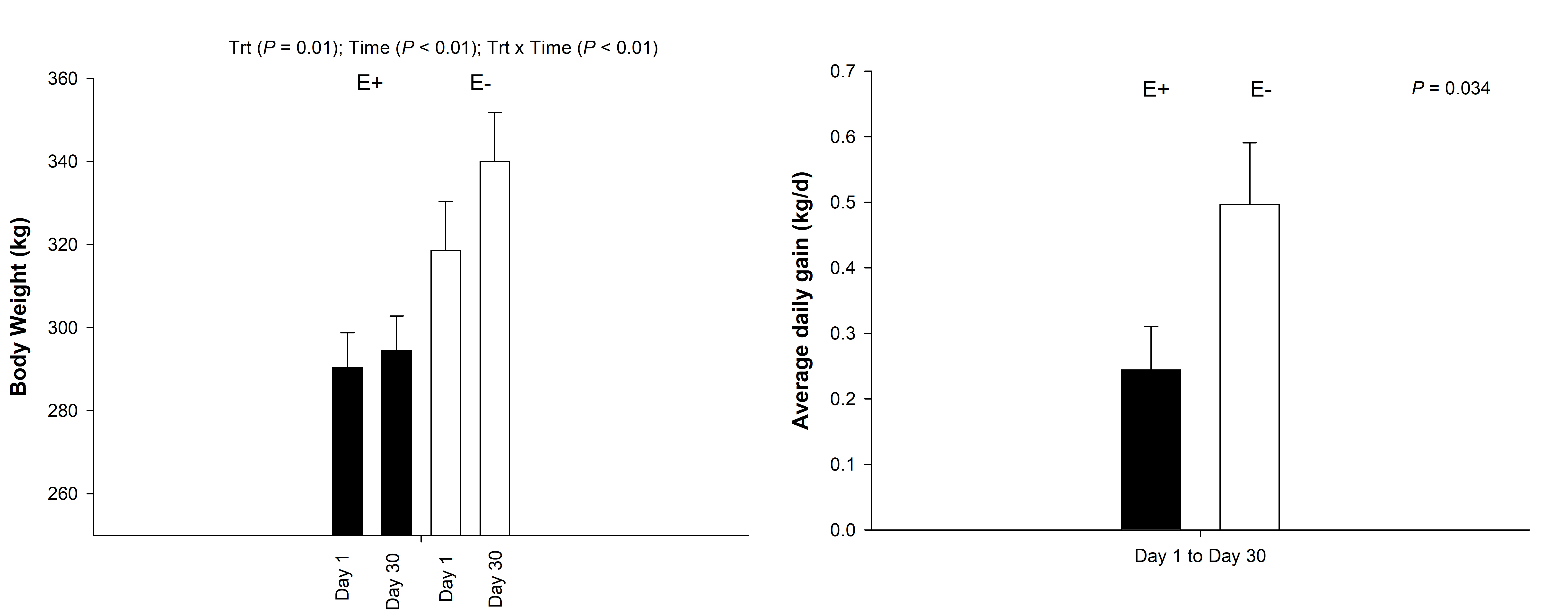

Supplement: S1 Fig — (TIF) [file pone.0306431.s010.tif]

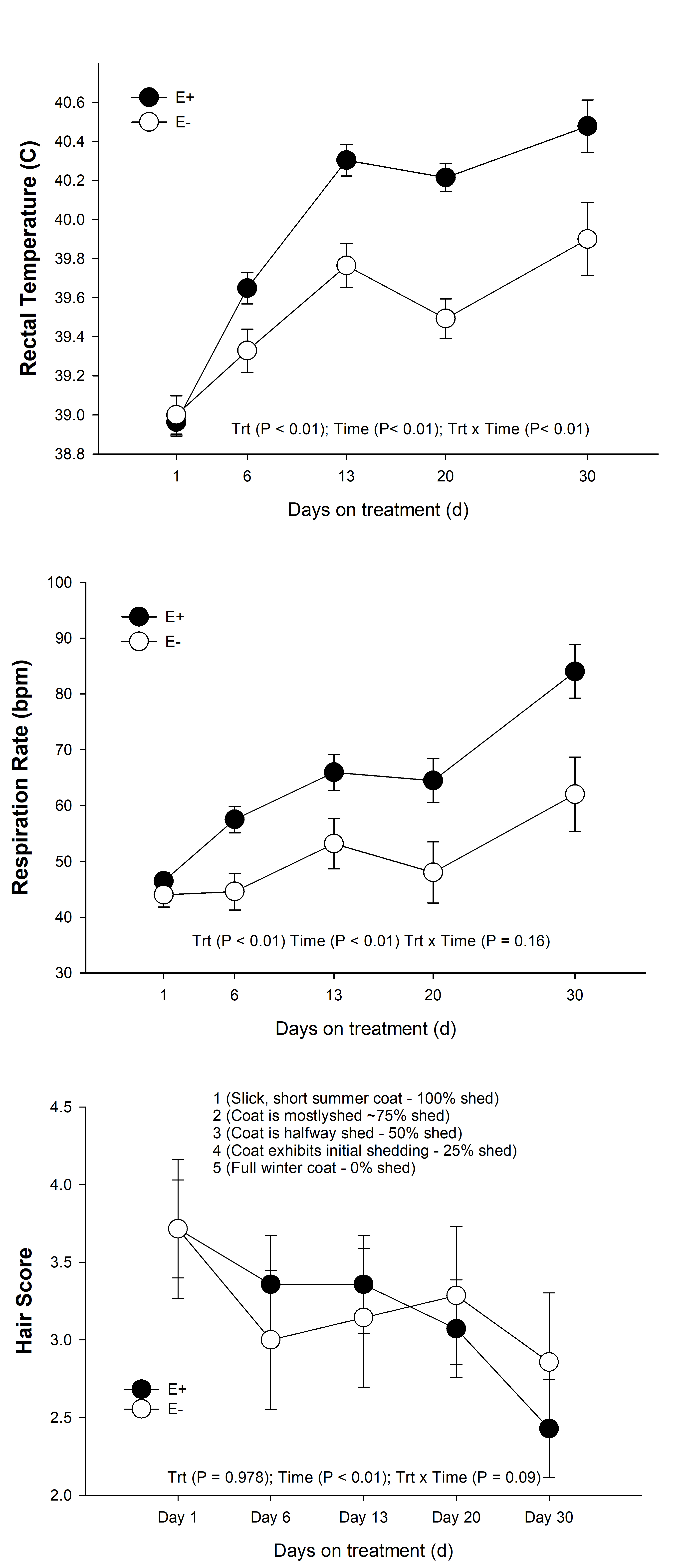

Supplement: S2 Fig — (TIF) [file pone.0306431.s011.tif]

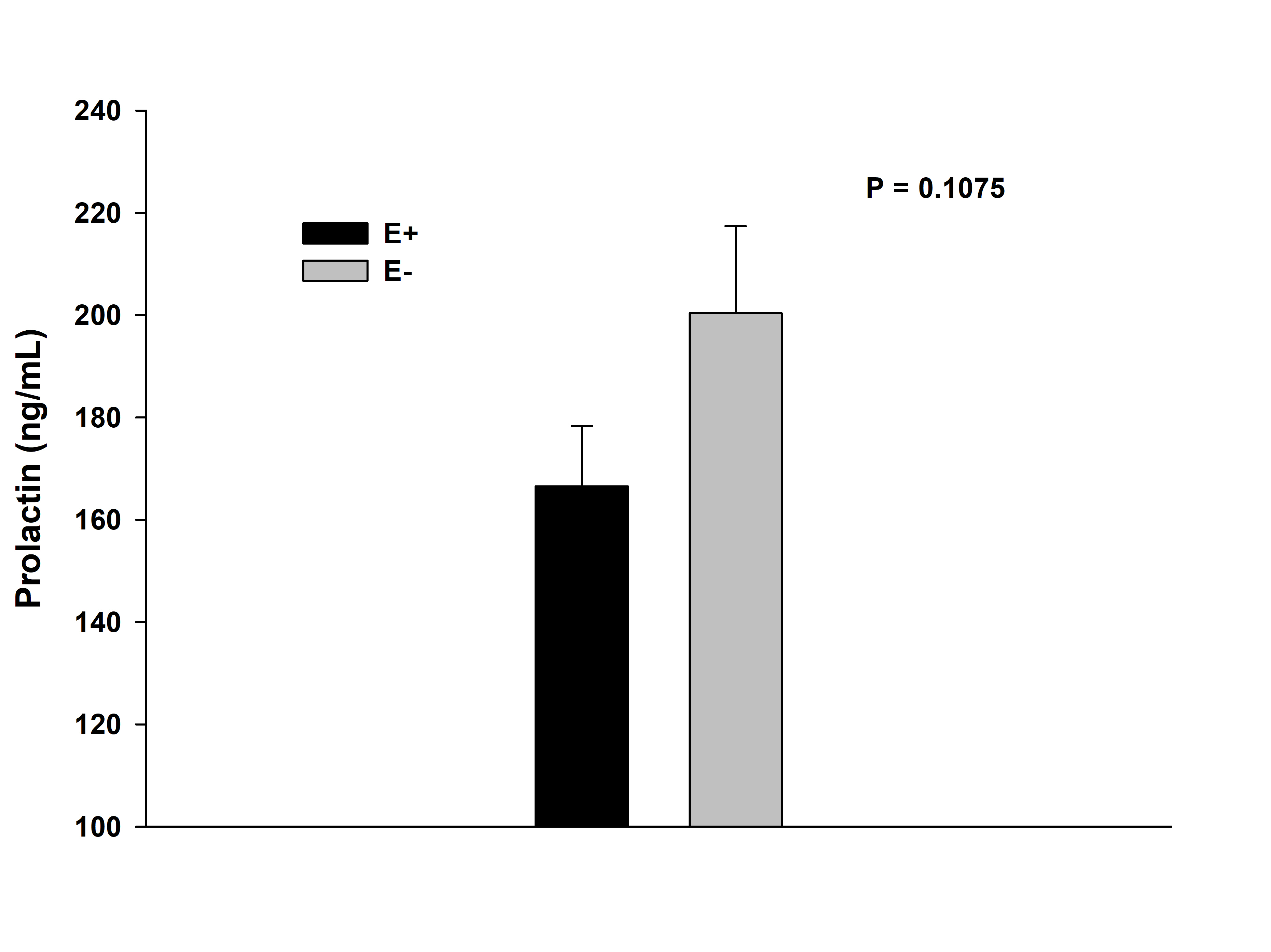

Supplement: S3 Fig — (TIF) [file pone.0306431.s012.tif]

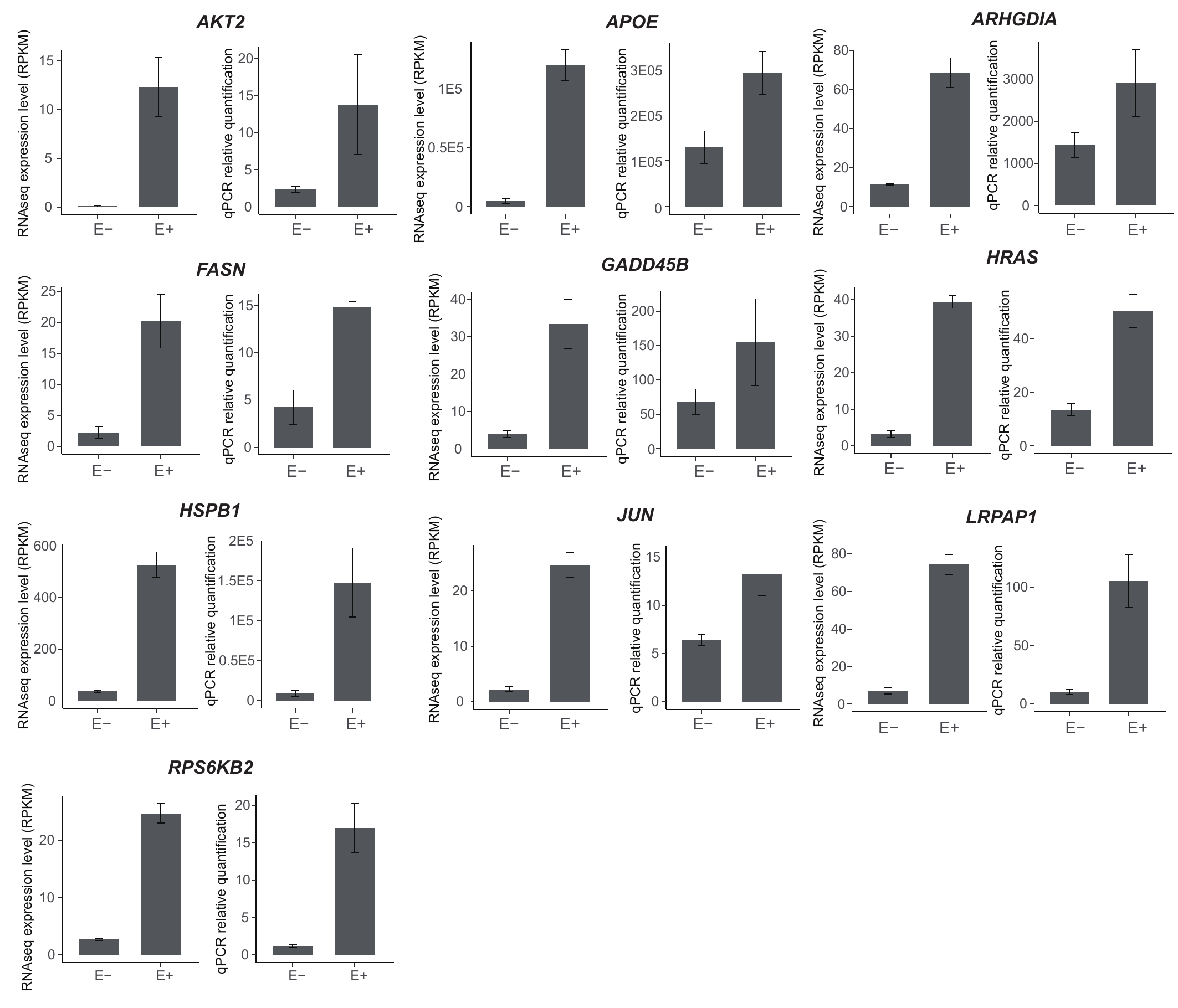

Supplement: S4 Fig — (TIFF) [file pone.0306431.s013.tiff]

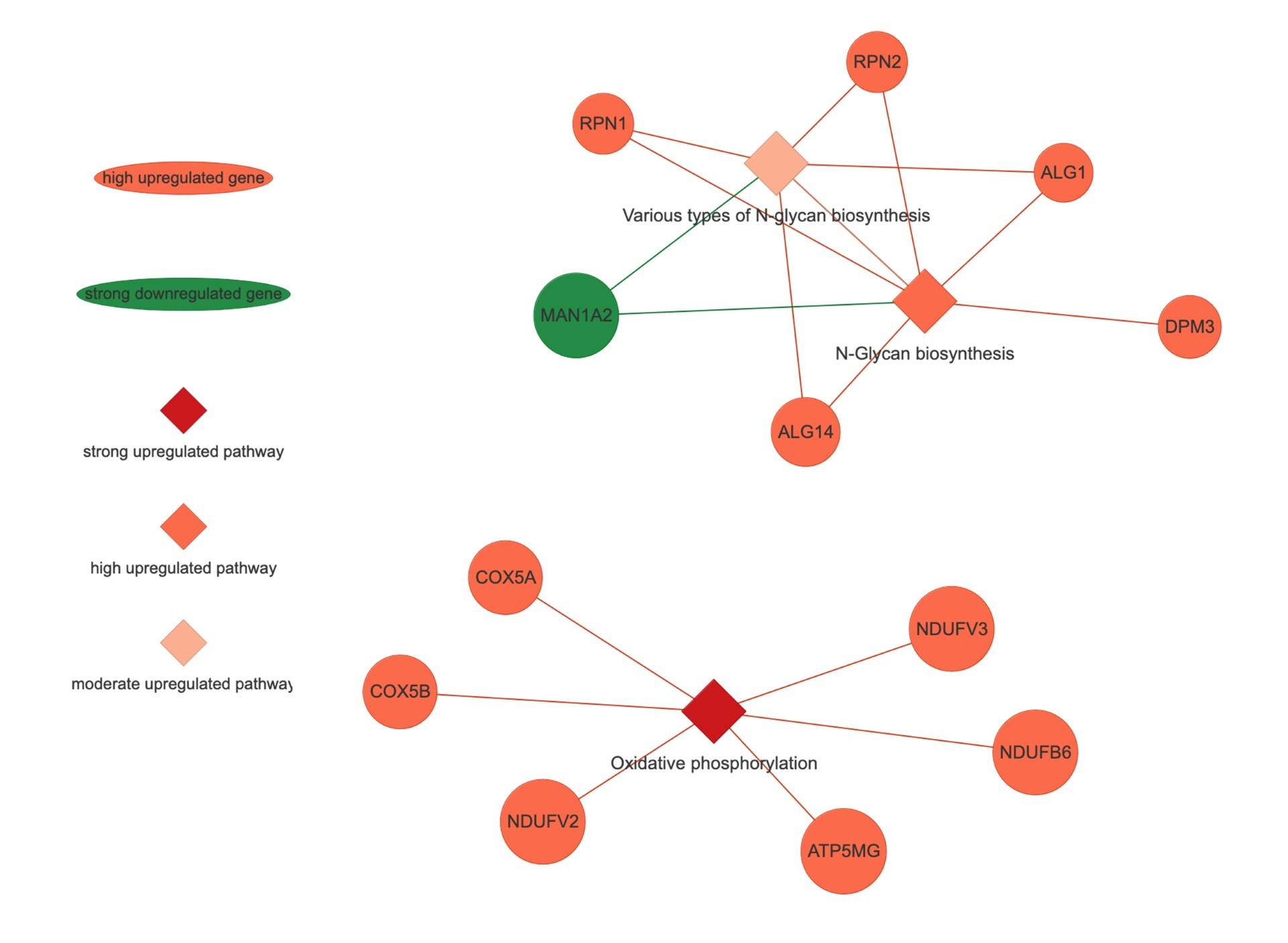

Supplement: S5 Fig — (TIFF) [file pone.0306431.s014.tiff]

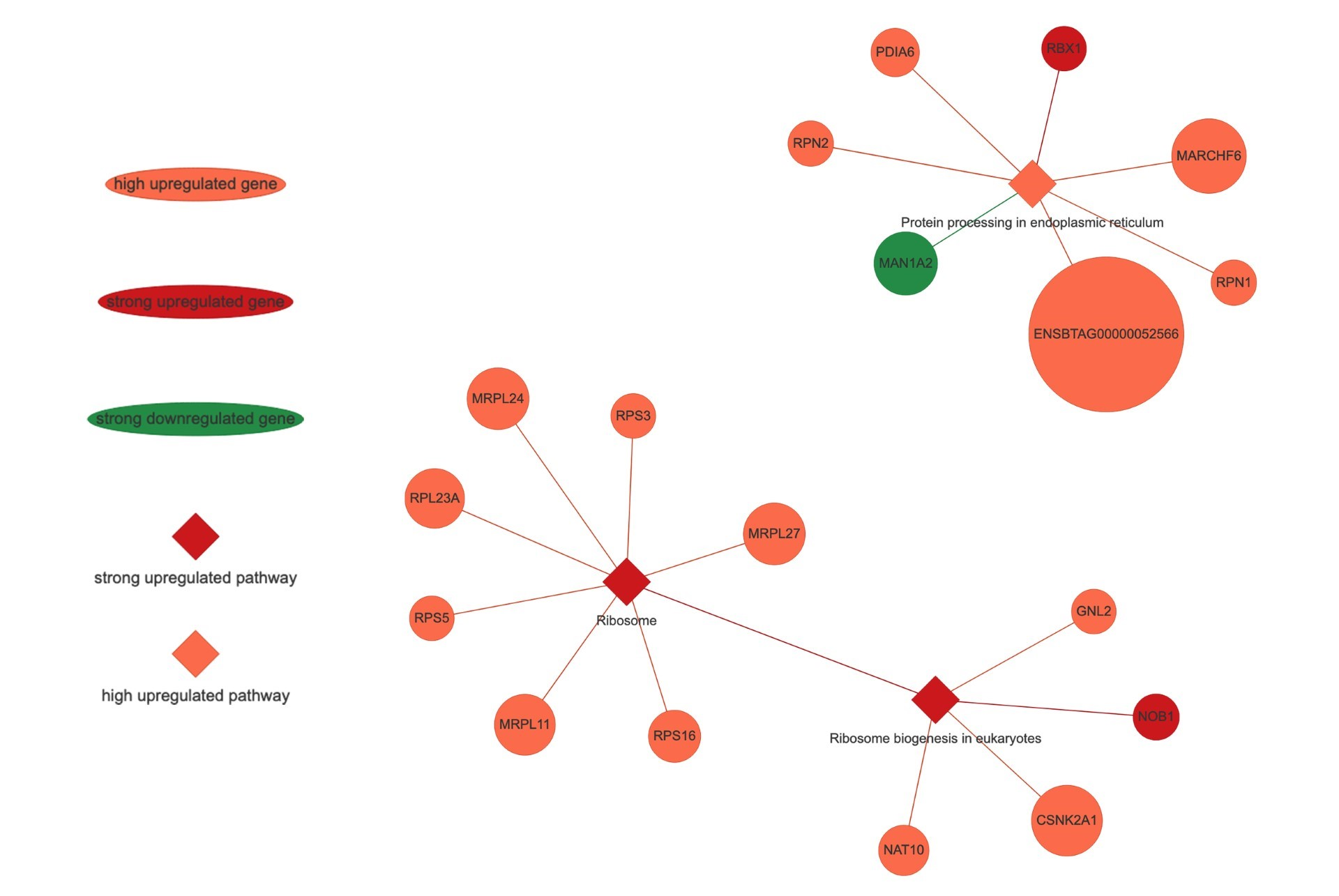

Supplement: S6 Fig — (TIFF) [file pone.0306431.s015.tiff]

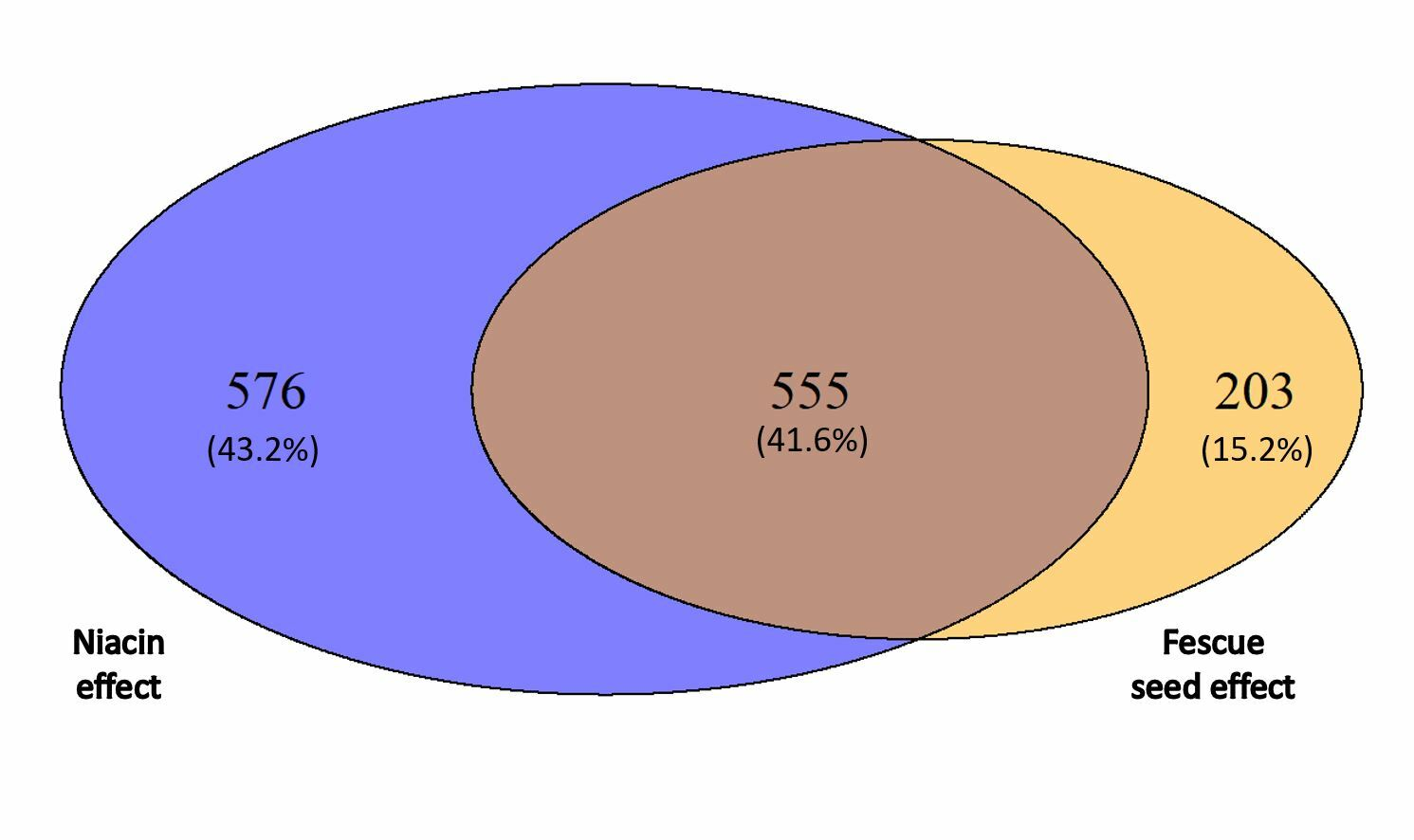

Supplement: S7 Fig — (TIFF) [file pone.0306431.s016.tiff]
